# Estimating the prevalence of overweight and obesity in Nigeria in 2020: a systematic review and meta-analysis

**DOI:** 10.1080/07853890.2021.1897665

**Published:** 2021-03-30

**Authors:** Davies Adeloye, Janet O. Ige-Elegbede, Martinsixtus Ezejimofor, Eyitayo O. Owolabi, Nnenna Ezeigwe, Chiamaka Omoyele, Rex G. Mpazanje, Mary T. Dewan, Emmanuel Agogo, Muktar A. Gadanya, Wondimagegnehu Alemu, Michael O. Harhay, Asa Auta, Akindele O. Adebiyi

**Affiliations:** a Centre for Global Health, Usher Institute, University of Edinburgh, Edinburgh, UK; b Centre for Public Health and Wellbeing, School of Health and Social Wellbeing, University of the West of England, Bristol, UK; c Division of Health Sciences, University of Warwick, Coventry, UK; d Clinical Standard Unit, British Association of Dermatologists (BAD), London, UK; e Centre for Global Surgery, Department of Global Health, Stellenbosch University, Tygerberg, South Africa; f Federal Ministry of Health, Abuja, Nigeria; g Nigeria Country Office, World Health Organization, Abuja, Nigeria; h Resolve to Save Lives, Abuja, Nigeria; i Department of Community Medicine, Aminu Kano Teaching Hospital, Bayero University, Kano, Nigeria; j International health Consultancy, Atlanta, GA, USA; k Department of Biostatistics, Epidemiology and Informatics, University of Pennsylvania Perelman School of Medicine, Philadelphia, PA, USA; l School of Pharmacy and Biomedical Sciences, University of Central Lancashire, Preston, UK; m College of Medicine, University of Ibadan, Ibadan, Nigeria

**Keywords:** Obesity, overweight, prevalence, non-communicable diseases, epidemiology, Nigeria

## Abstract

**Background:**

Targeted public health response to obesity in Nigeria is relatively low due to limited epidemiologic understanding. We aimed to estimate nationwide and sub-national prevalence of overweight and obesity in the adult Nigerian population.

**Methods:**

MEDLINE, EMBASE, Global Health, and Africa Journals Online were systematically searched for relevant epidemiologic studies in Nigeria published on or after 01 January 1990. We assessed quality of studies and conducted a random-effects meta-analysis on extracted crude prevalence rates. Using a meta-regression model, we estimated the number of overweight and obese persons in Nigeria in the year 2020.

**Results:**

From 35 studies (*n* = 52,816), the pooled crude prevalence rates of overweight and obesity in Nigeria were 25.0% (95% confidence interval, CI: 20.4–29.6) and 14.3% (95% CI: 12.0–15.5), respectively. The prevalence in women was higher compared to men at 25.5% (95% CI: 17.1–34.0) versus 25.2% (95% CI: 18.0–32.4) for overweight, and 19.8% (95% CI: 3.9–25.6) versus 12.9% (95% CI: 9.1–16.7) for obesity, respectively. The pooled mean body mass index (BMI) and waist circumference were 25.6 kg/m^2^ and 86.5 cm, respectively. We estimated that there were 21 million and 12 million overweight and obese persons in the Nigerian population aged 15 years or more in 2020, accounting for an age-adjusted prevalence of 20.3% and 11.6%, respectively. The prevalence rates of overweight and obesity were consistently higher among urban dwellers (27.2% and 14.4%) compared to rural dwellers (16.4% and 12.1%).

**Conclusions:**

Our findings suggest a high prevalence of overweight and obesity in Nigeria. This is marked in urban Nigeria and among women, which may in part be due to widespread sedentary lifestyles and a surge in processed food outlets, largely reflective of a trend across many African settings.

**KEY MESSAGES**
- About 12 million persons in Nigeria were estimated to be obese in 2020, with prevalence considerably higher among women. Nutritional and epidemiological transitions driven by demographic changes, rising income, urbanization, unhealthy lifestyles, and consumption of highly processed diets appear to be driving an obesity epidemic in the country.

## Introduction

The disease burden from overweight and obesity has continued to increase globally [1]. The World Health Organization (WHO) reported that overweight and obese persons nearly tripled between 1975 and 2016 [1]. Recently, Ng et al. [2] reported that the prevalence of overweight and obesity increased significantly worldwide in children and young adults between 1980 and 2013 [2]. In 2016, more than 1.9 billion adults aged 18 years or more were overweight, with 650 million obese [1]. Obesity and overweight are strongly linked with several cardio-metabolic disorders including high blood pressure, high blood glucose, insulin resistance, high blood cholesterols, coronary heart disease, stroke and cancers [3]. These are important contributors to poor health outcomes, particularly for many cases of COVID-19 in African population groups. Globally, over 3 million deaths and an estimated 36 million DALYs were attributed to overweight and obesity annually [1,2].

Although, previously thought to be challenges in high-income settings, current trends reveal overweight and obesity are on the rise across urban settings in several low- and middle-income countries (LMICs) [2]. While a plateauing in obesity prevalence have been recorded since the mid-2000s in many high-income countries, prevalence rates have been increasing rapidly in LMICs, including several African countries, over the same period [4]. In sub-Saharan Africa, about 30% and 10% of adults are overweight and obese, respectively [4,5]. In Nigeria, nutritional and epidemiological transitions driven by demographic changes, rising income, urbanization, unhealthy lifestyles, and consumption of highly processed diets are among the leading contributors to overweight and obesity [5,6]. In fact, the burden has extended to younger population age groups in the country with about 9% of children aged 5–9 years estimated to be obese or overweight [6,7]. Recent evidence on the high burden of cardiovascular disease [8,9], diabetes mellitus [10] and hypertension [11] in Nigeria mirrors the classic population pyramid that depicts a greater proportion of younger population with increased vulnerability [12]. These chronic conditions have also been linked to the clustering of major risk factors in many epidemiological studies [8–10,13], with overweight and obesity being the common denominators. Obesity and related co-morbidities have greatly impacted on individuals’ health, self-esteem, educational attainment, quality of life and overall productivity [7].

Ng et al. [2] noted that obesity is not only increasing globally, there are also relatively no national success stories on its prevention in the past three decades from several countries, necessitating urgent global action to help countries effectively intervene. Recently, the WHO member nations, including Nigeria, have targeted halting the rise in obesity by 2025 [7]. Since then, there have been widespread in-country calls for regular monitoring of changes in overweight and obesity prevalence across populations, albeit affected by a dearth of data and information on the prevalence of overweight and obesity in Nigeria. Despite some emerging reports in recent times, gaps still exist in the understanding of nationwide predictors of overweight and obesity particularly in the adult population [10,14]. This study, therefore, aims to estimate nation-wide and zonal (sub-national) prevalence of obesity and overweight in the adult Nigerian population. This would be essential to quantify health effects and prompt decision-makers to prioritize relevant actions.

## Methods

The study was conducted in compliance with the PRISMA guidelines [15].

### Search strategy

Databases searched include MEDLINE, EMBASE, Global Health, and Africa Journals Online (AJOL). At this stage, we broadly searched for studies on overweight and/or obesity in Nigeria (see search terms in Table 1). Searches were conducted on 01 July 2020 and limited to studies published after 01 January 1990. Unpublished documents were sourced from Google Scholar and Google searches. Titles and abstracts of studies were reviewed, and full-texts of relevant studies accessed (see Selection criteria). References of accessed full-texts were further hand-searched for additional studies. Authors of selected papers were contacted for any missing information on study characteristics and prevalence estimates.

**Table 1.** Search terms.

Number	Searches
1	africa/ or africa, sub-sahara/ or africa, western/ or nigeria/
2	exp vital statistics/
3	(incidence* or prevalence* or morbidity or mortality).tw.
4	(disease adj3 burden).tw.
5	exp “cost of illness”/
6	case fatality rate.tw
7	hospital admissions.tw
8	Disability adjusted life years.mp.
9	(initial adj2 burden).tw.
10	exp risk factors/
11	2 or 3 or 4 or 5 or 6 or 7 or 8 or 9 or 10
12	exp obesity / or obese/ or overweight/ or high bmi or waist circumference
13	1 and 11 and 12
14	Limit 13 to “1990-current”

### Selection criteria

Population- or community-based studies reporting on the prevalence of overweight and/or obesity in a Nigerian setting were selected. We also selected studies on cardio-metabolic risks and extracted data on overweight and obesity from such studies when reported. We excluded hospital-based reports, studies on Nigerians in diaspora, reviews, editorials, view-points and commentaries.

### Case definitions

We checked for definition of overweight and obesity in selected studies, with both broadly identified as abnormal or excessive fat accumulation presenting a risk to health [1]. For analysis, we ensured studies employed crude population measure of obesity using body mass index (BMI), equivalent to a person’s weight (in kilograms, *kg*) divided by the square of his or her height (in metres, *m*). A person is considered obese if the BMI is 30 or higher, while a BMI of 25 or higher is considered overweight.

### Data extraction

Assessment of eligible studies was conducted independently by two reviewers – DA (PhD) and AA (PhD) – with an eligibility guideline based on the selection criteria to ensure consistency. Disagreements in study selection were resolved by consensus. We extracted data on the study location (including geo-political zones), period, design, setting (urban or rural), sample size, diagnostic criteria and mean age of sample population. These were matched with corresponding data on overweight or obese persons and respective prevalence rates reported in each study. For multiple studies reporting data from the same study site, population or cohort, the first published study was selected, and all additional data from the other studies were extracted and merged with data from the selected paper.

### Quality assessment

JOI (MSc) and EOO (PhD) independently assessed quality of selected studies with disagreements resolved in another meeting with DA. Adapting a validated quality assessment guideline for studies on epidemiology of chronic diseases [16,17], already used in previous studies [10], we based our grading on three broad criteria. These include: (i) design – appropriate approach to statistical analysis with limitations sufficiently described, (ii) identification of cases – case ascertainment using standard or acceptable guideline or protocol, and (iii) sampling – appropriate approach to sampling representative of the larger population of the study location, e.g. the town, city or local government area. Studies were finally graded as *high, moderate, or low quality* (see Tables 2 and 3 for details of all full-text manuscripts accessed and quality grading).

**Table 2.** Approach to quality assessment.

Quality criteria	Assessment	Score	Maximum score
Sampling (was it described and representative of a target subnational population?)	Yes	2	2
	Not representative	1	
	Not described	0	
Appropriateness of statistical analysis	Yes	1	1
	No	0	
Case ascertainment (was the procedure for identification of cases clearly described?)	Yes	2	2
	Ambiguous	1	
	Not described	0	
Total [high (4–5), moderate (2–3), or low quality (0–1)]			5

**Table 3.** Characteristics of studies on prevalence of overweight or obesity in Nigeria.

First author	Study period	Location	Geopolitical zone	Study design	Study setting	Quality
1. Abegunde [20]	2011	Oyo State	South-west	Descriptive cross-sectional study	Mixed	High
2. Agaba [21]	2014	Jos, Plateau State	North-central	Descriptive cross-sectional study	Urban	High
3. Akinbodewa [22]	2014	Akure & Ondo, Ondo State	South-west	Descriptive cross-sectional study	Mixed	High
4. Emerole [23]	2007	Owerri, Imo State	South-east	Descriptive cross-sectional study	Urban	Moderate
5. Ibekwe [24]	2012	Oghara, Delta State	South-south	Descriptive cross-sectional study	Rural	Moderate
6. Odey [25]	2011	Calabar, Cross River State	South-south	Descriptive cross-sectional study	Urban	Moderate
7. Odugbemi [26]	2010	Tejuosho, Lagos	South-west	Descriptive cross-sectional study	Urban	High
8. Lawoyin [27]	1998	Idikan Ibadan, Oyo State	South-west	Population-based cross-sectional study	Rural	Moderate
9. Ugwuja [28]	2008	Abakaliki, Ebonyi State	South-east	Descriptive cross-sectional study	Urban	High
10. Oladapo [29]	2005	Egbeda, Oyo State	South-west	Descriptive cross-sectional study	Rural	High
11. Okaka [30]	2013	O*via*, Edo state	South-south	Population-based cross-sectional study	Rural	High
12. Odenigbo [31]	2008	Asaba, Delta State	South-south	Population-based cross-sectional study	Semi-urban	High
13. Okagua [32]	2016	Port-Harcourt, Rivers State	South-south	Population-based cross-sectional study	Urban	Moderate
14. Adesina [33]	2010	Port-Harcourt, Rivers State	South-south	Population-based cross-sectional study	Urban	Moderate
15. Oyeyemi [34]	2013	Maiduguri, Yobe State	North-east	Population-based cross-sectional study	Semi-urban	High
16. Iwuala [35]	2014	Lagos State	South-west	Descriptive cross-sectional study	Urban	High
17. Musa [36]	2012	Benue State	North-central	Descriptive cross-sectional study	Mixed	Moderate
18. Yusuf [37]	2013	Kano State	North-west	Descriptive cross-sectional study	Urban	High
19. Odunaiya [38]	2010	Ibadan, Oyo State	South-west	Population-based cross-sectional study	Urban	High
20. Ezejimofor [39]	2014	Niger Delta, Delta State	South-south	Community-based cross-sectional study	Rural	High
21. Ojji [40]	2010	Abuja, FCT	North-central	Prospective cohort study	Urban	High
22. Akintunde [41]	2010	Osogbo, Osun State	South-west	Population-based cross-sectional study	Mixed	High
23. Akpan [42]	2015	Akwa Ibom States	South-south	Population-based cross-sectional study	Urban	High
24. Chukwuonye [43]	2013	Abia State	South-east	Population-based house-to-house survey	Mixed	High
25. Ezekwesili [44]	2016	Anambra State	South-east	Population-based cross-sectional study	Mixed	High
26. Iloh [45]	2009	Imo State	South-east	Descriptive cross-sectional study	Rural	High
27. Iloh [46]	2008	Imo State	South-east	Descriptive cross-sectional study	Rural	Moderate
28. Iloh [47]	2010	Owerri, Imo State	South-east	Descriptive cross-sectional study	Urban	Moderate
29. Murthy [48]	2013	National	National	Population-based cross-sectional study	Mixed	High
30. Okafor [49]	2014	Enugu, Enugu State	South-east	Population-based cross-sectional study	Urban	Moderate
31. Ogah [50]	2012	Umuahia, Abia State	South-east	Population-based cross-sectional study	Mixed	High
32. Olamoyegun [51]	2016	Ekiti State	South-west	Population-based cross-sectional study	Semi-urban	Moderate
33. Shittu [52]	2017	Oke Ogun, Oyo State	South-west	Population-based cross-sectional study	Rural	Moderate
34. Suleiman [53]	2011	Amassoma, Bayelsa State	South-south	Descriptive cross-sectional study	Semi-urban	Moderate
35. Wahab [54]	2006	Katsina, Katsina State	North-west	Population-based cross-sectional study	Urban	High

### Data analysis

We first conducted a random-effects meta-analysis, using the DerSimonian and Laird Method [18], on the individual study estimates to generate crude national and regional pooled estimates of the prevalence of obesity or overweight in Nigeria. We estimated standard errors from individual study prevalence and population denominators, assuming a binomial (or Poisson) distribution. Heterogeneity between studies was assessed using I-squared (*I^2^*) statistics. Subgroup analysis (based on regions and settings) was conducted to explore sources of heterogeneity. We investigated publication bias by conducting an Egger’s test and visual inspecting a Funnel plot of the logarithm of obesity prevalence and its standard error. A meta-regression epidemiologic model accounting for study sample size, study period, and age was constructed to determine prevalence distribution of overweight and obesity by age of the Nigerian population. From the age-adjusted prevalence rates, we estimate the absolute number of overweight and obese persons in Nigeria at midpoints of the United Nation (UN) population 5-year age groups for Nigeria for the year 2020 [19]. This approach to data analysis has been employed in previous studies [10,11]. All statistical analyses were conducted on STATA V.14 (Stata Corp, College Station, TX, USA).

## Results

### Search results

**Figure 1.**
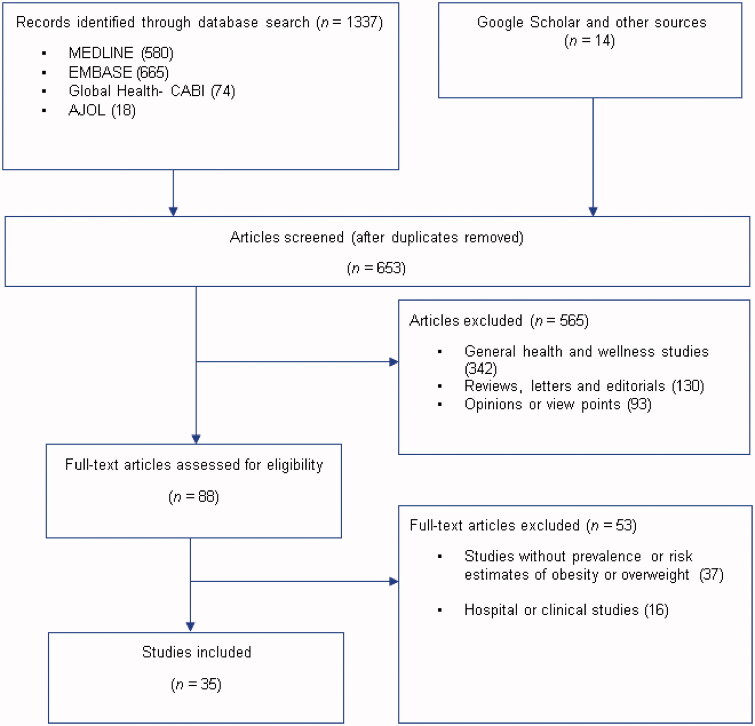
Flow chart of selection of studies on obesity or overweight in Nigeria.

A total of 1337 studies were retrieved from the databases – MEDLINE 580, EMBASE 665, Global Health 74, and AJOL 18. Additional 14 studies were identified through Google Scholar, Google searches, and hand-searching reference lists of relevant studies. After duplicates have been removed, 653 titles were screened for relevance (i.e. any population- or community-based studies on overweight or obesity in Nigeria). On applying the selection criteria, 565 studies were excluded. Hence, 88 full-texts assessed which were screened explicitly using the selection and quality criteria. Thirty-five studies [20–54] were selected for the review (Figure 1).

### Study characteristics

The 35 studies spread across the southern and northern parts of Nigeria (Table 3). South-south had the highest output with 10 studies, closely followed by South-east and South-west with nine studies each. Three studies were retrieved from the North-central, two from the North-west and one from the North-east. Most studies (*n* = 19) were conducted in urban settings, nine in rural and seven in mixed urban-rural settings. Twenty-two studies were rated as high quality, with the remaining 13 rated as moderate quality. Study period ranged from 1993 to 2017, with most studies conducted within a one-year period. There were 56 data-points extracted from all studies, covering a population of 52,816, with mean age ranging from 14.7 to 61.7 years (Table 3). Heterogeneity was high across studies, with I-squared (*I^2^*) mostly above 99.0% (*p* < .001) across different settings. When sub-groups (regions and settings) were separately considered, our data returned highest heterogeneity from the North-central (99.7%), North-west (99.7%) and rural settings (99.8%). The Funnel plot suggests some degree of publication bias with large studies reporting high prevalence rates mainly published. The Egger’s test confirms presence of small study effects (*p* < .001) (Supplementary material).

### Prevalence of overweight in Nigeria

**Figure 2.**
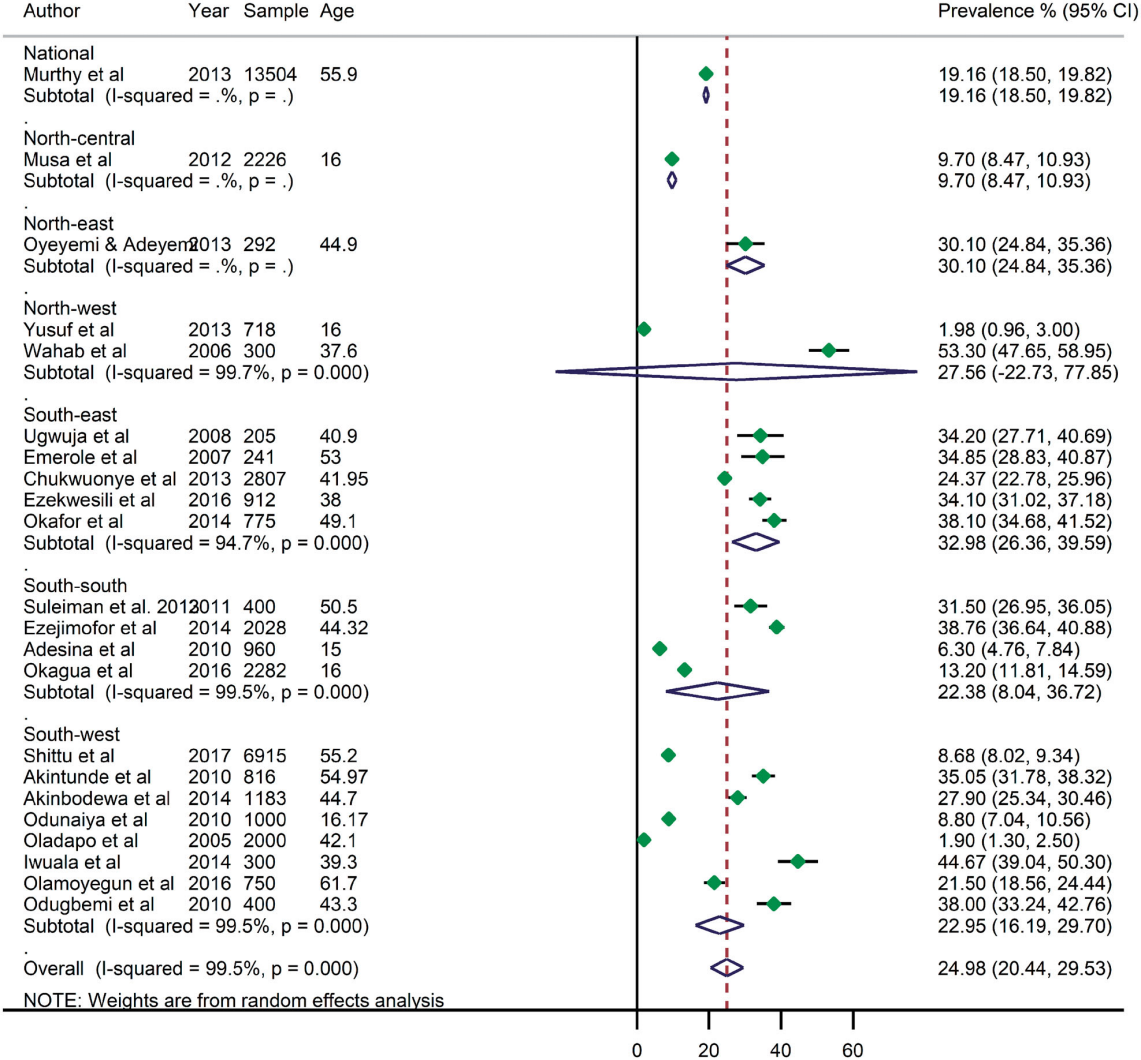
Crude prevalence rate of overweight in Nigeria, by geopolitical zones.

The prevalence of overweight varies widely across different settings in Nigeria, ranging from 1.9% in Egbeda, Oyo State, a rural setting in South-west Nigeria [29] to 53.3% in Katsina, North-west Nigeria [54]. From all data points, the pooled crude prevalence of overweight persons in Nigeria was 25.0% (95% CI: 20.4–29.6) (Figure 2). The prevalence in women was slightly higher at 25.5% (17.1–34.0) compared to men at 25.2% (18.0–32.4) (Table 4, Supplementary material). The prevalence was highest in South-east (33.0%, 26.4–40.0). Although, both the North-east and North-west had limited datapoints, the prevalence of overweight in both regions was also high at 30.1% (24.5–35.3) and 27.6% (22.7–77.9), respectively. The South-west and South-east had relatively similar rates at 23.0% (16.2–29.7) and 22.4% (8.0–36.7), respectively. The prevalence of overweight persons was higher among urban dwellers (27.2%, 20.1–34.3) compared to rural settings (16.4%, 4.7–28.1) (Table 4).

**Table 4.** Pooled crude estimates of prevalence of overweight and obesity in Nigeria, by sub-groups.

	Both sexes		Men		Women	
	Prevalence % (95% CI)	I^2^ %, *p* value	Prevalence % (95% CI)	I^2^ %, *p* value	Prevalence % (95% CI)	I^2^ %, *p* value
Nation-wide						
Overweight	25.0 (20.4–29.5)	99.5, .000	25.2 (18.0–32.4)	99.0, .000	25.5 (17.1–34.0)	99.2, .000
Obesity	14.3 (12.0–16.5)	99.2, .000	12.9 (9.1–16.7)	98.7, .000	19.8 (13.9–25.6)	99.2, .000
Geopolitical zone						
North-central						
Overweight	9.7 (8.5–10.9)	–	–	94.8, .000	–	–
Obesity	18.5 (1.4–38.3)	99.7, .000	14.4 (6.9–21.8)	93.4, .000	42.0 (26.5–57.5)	96.0, .000
North-east						
Overweight	30.1 (24.5–35.3)	–	36.3 (29.5–43.1)	–	26.8 (18.2–35.0)	–
Obesity	24.0 (19.1–28.9)	–	42.1 (35.1–49.1)	–	14.3 (7.5–21.1)	–
North-west						
Overweight	27.6 (22.7–77.9)	99.7, .000	21.4 (18.4–61.6)	98.8, .000	32.1 (26.3–90.5)	99.6, .000
Obesity	10.8 (8.9–30.5)	98.6, .000	4.6 (3.8–13.1)	91.0, .000	15.8 (13.1–43.4)	98.5, .000
South-east						
Overweight	33.0 (26.4–40.0)	94.7, .000	32.7 (22.7–42.6)	90.5, .000	30.3 (19.0–41.8)	95.0, .000
Obesity	13.6 (8.4–18.8)	99.3, .000	15.4 (2.7–28.2)	98.8, .000	20.5 (7.2–33.8)	98.9, .000
South-south						
Overweight	22.4 (8.0–36.7)	99.5, .000	3.7 (2.0–5.4)	–	9.4 (6.8–12.0)	–
Obesity	13.6 (9.4–17.8)	98.3, .000	2.6 (0.9–6.0)	85.6, .000	8.5 (2.1–14.9)	94.1, .000
South-west						
Overweight	23.0 (16.2–29.7)	99.5, .000	25.2 (3.7–467)	99.1, .000	23.0 (4.6–41.4)	92.6, .000
Obesity	14.9 (9.6–20.1)	99.4, .000	12.3 (3.0–21.5)	98.8, .000	16.8 (5.4–28.2)	99.3, .000
Settings						
Urban						
Overweight	27.2 (20.1–34.3)	99.1, .000	26.9 (17.4–36.4)	98.4, .000	28.1 (15.6–40.5)	98.9, .000
Obesity	14.4 (11.1–17.7)	98.9, .000	10.9 (7.3–14.5)	97.3, .000	18.5 (11.7–25.2)	98.7, .000
Rural						
Overweight	16.4 (4.7–28.1)	99.8, .000	1.9 (0.9–2.8)	–	1.8 (1.0–2.6)	–
Obesity	12.1 (8.5–15.8)	99.1, .000	14.0 (10.5–38.5)	99.6, .000	13.4 (8.7–36.4)	99.7, .000
Mixed						
Overweight	24.9 (18.6–31.2)	99.0, .000	31.1 (17.6–44.6)	96.3, .000	27.8 (20.6–35.1)	61.3, .004
Obesity	16.7 (10.3–23.1)	99.6, .000	18.2 (3.2–33.3)	99.2, .000	28.6 (11.8–45.0)	87.6, .000

### Prevalence of obesity in Nigeria

**Figure 3.**
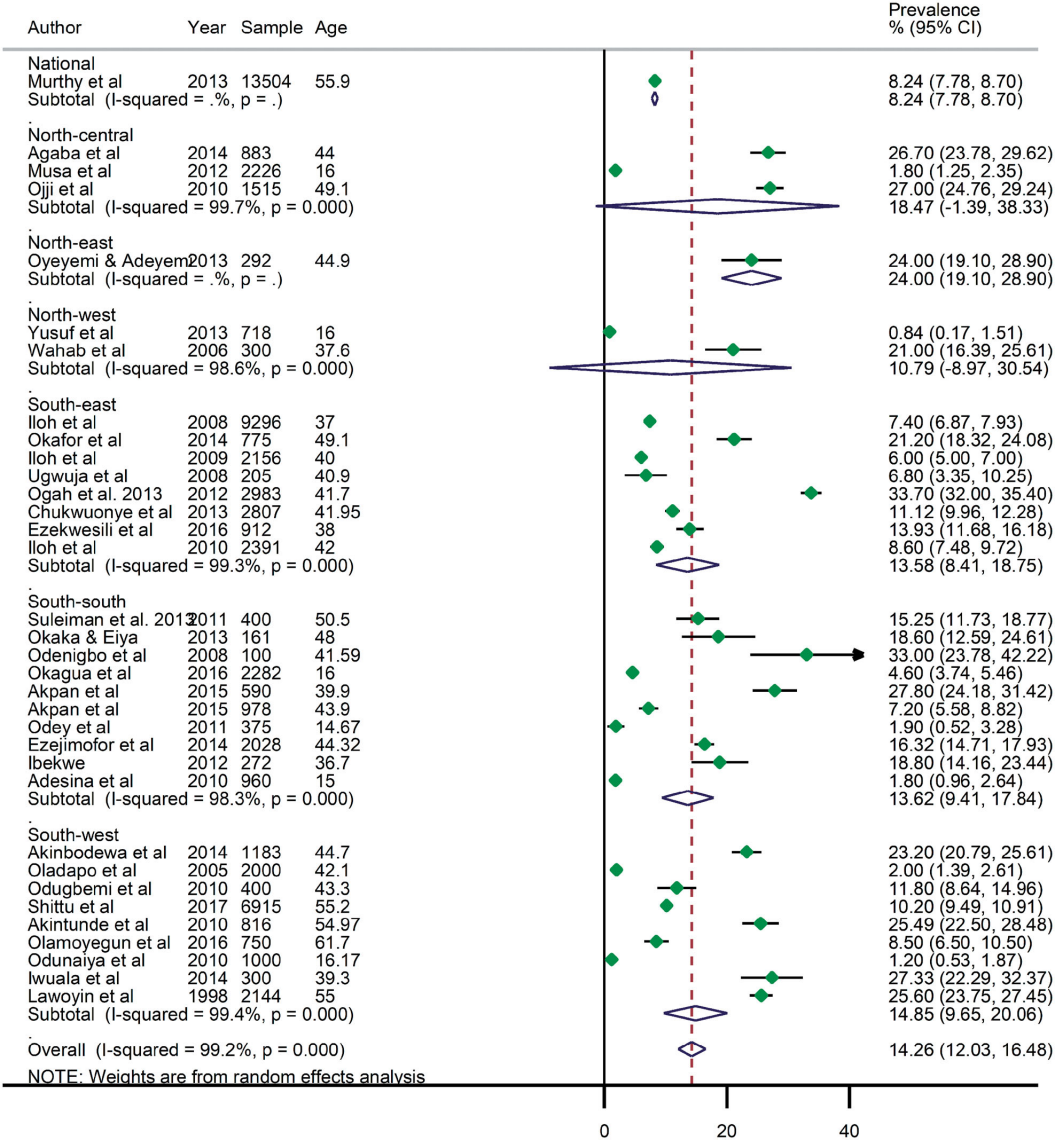
Crude prevalence rate of obesity in Nigeria, by geopolitical zones.

From all studies, the highest prevalence of obesity was reported in Umuahia, Abia State, South-east Nigeria in 2012 at 33.7% [50], with the lowest rate recorded in Kano, North-west Nigeria in 2013 at 0.84% [37]. The pooled (from all data points) crude prevalence of obesity in Nigeria was 14.3 (95% CI: 12.0–15.5) (Figure 3). As observed among overweight persons, the prevalence was higher among women (19.8%, 13.9–25.6) compared to the pooled rate in men (12.9%, 9.1–16.7) (Table 4, Supplementary material). Across geopolitical zones, the highest prevalence was in the North-east at 24.0 (19.1–28.9), although this was mainly from a single survey in the region. However, the North-central recorded a high pooled rate of obesity at 18.5% (1.4–38.3), while the North-west had the lowest rate at 10.8% (8.9–30.5). While the estimates from the Northern regions are marked by wide uncertainty intervals and may still be subject to further validation, the high estimates of obesity (and overweight) in at least two regions call for some public health concerns in these settings. Meanwhile, the Southern regions had nearly similar rates of obesity with the South-west at 14.9% (9.6–20.1), South-east 13.6% (8.4–18.8) and South-south 13.6% (9.4–17.8). The prevalence of obesity was higher in urban settings at 14.4% (11.1–17.7) compared to rural settings at 12.1% (8.5–15.8) (Table 4).

### Pooled mean BMI and waist circumference in Nigeria

**Figure 4.**
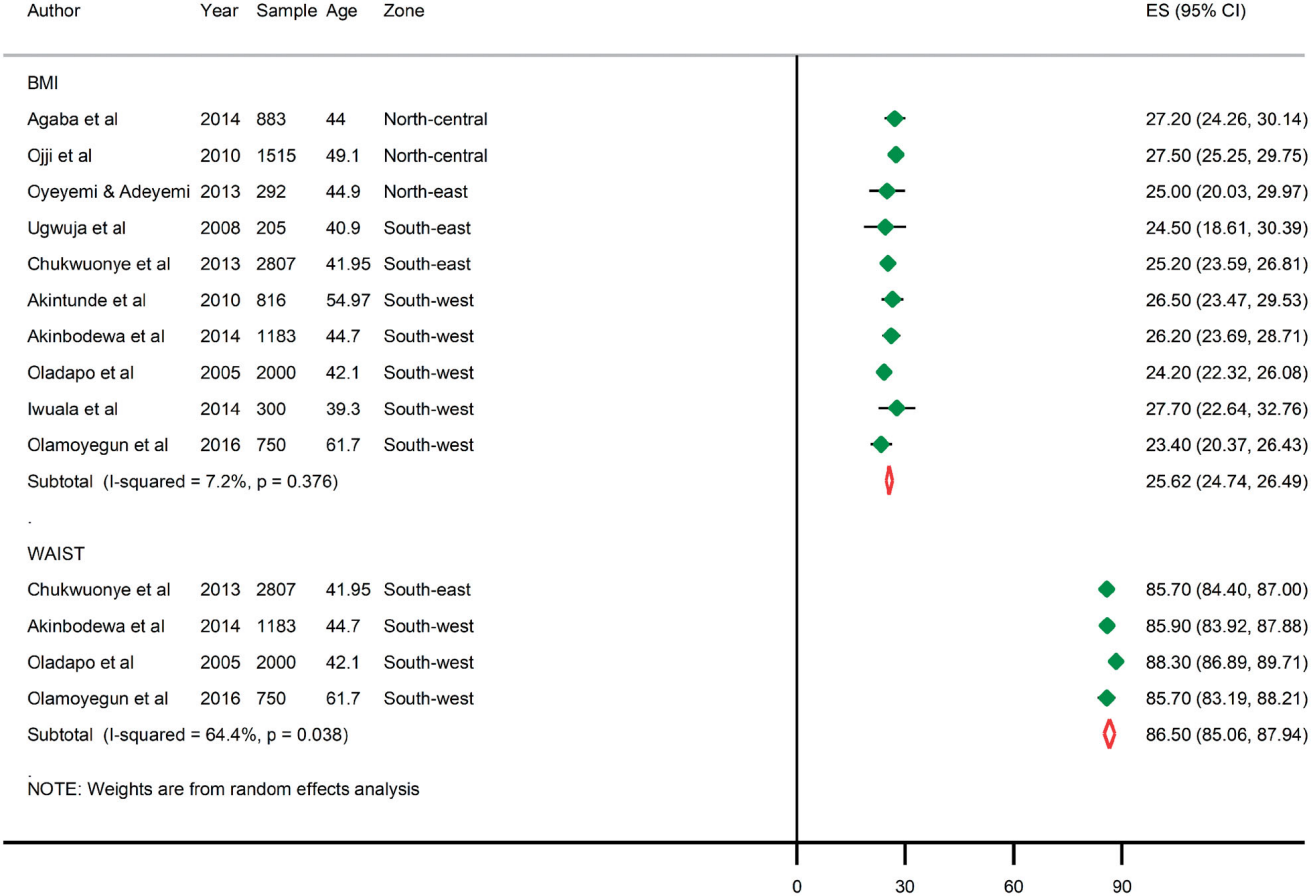
Pooled mean BMI and waist circumference in Nigeria. Note: BMI (kg/m^2^), waist circumference (cm).

From individual study estimates, the mean population BMI ranged from being normal (23.4 kg/m^2^) recorded in a rural setting in Ekiti State, South-west Nigeria [51], to an overweight population (27.7 kg/m^2^) recorded in the urban metropolis of Lagos State, also in the South-west [35]. The mean population waist circumference has a narrow margin, ranging from 85.7 centimetres (cm) recorded in Abia State, South-east Nigeria [43] to 88.3 cm measured in Egbeda Oyo State, South-west Nigeria [29]. From all data-points, the pooled mean BMI in Nigeria was 25.6 kg/m^2^ and the mean waist circumference was 86.5 cm, which both suggest that several persons may be slightly overweight in the country (Figure 4).

### Estimated number of overweight or obese persons in Nigeria

The meta-regression epidemiologic modelling, adjusted for study period and sample size, was applied to mean ages and crude prevalence rates of overweight and obesity extracted from individual studies (Supplementary material). Although advancing age was a significant variable, year of study was not, so we did not conduct any trend analyses. Using the United Nations demographic projections for Nigeria, the absolute number of overweight persons among persons aged 15 years or more in the country was 20.9 million in 2020, with an age-adjusted prevalence of 20.3% (Table 5). In the same year, obese persons in Nigeria were estimated at 12 million, accounting for 11.6% among persons age 15 years or more (Table 5).

**Table 5.** Absolute number of overweight and obese persons in Nigeria, aged 15 years or more in 2020.

Age (years)	Overweight			Obese		
	Prevalence (%)	Population (000)	Cases (000)	Prevalence (%)	Population (000)	Cases (000)
*15–19*	12.0	18,603.868	2229.674	4.7	18,603.868	871.777
20–24	14.3	15,981.820	2292.592	6.7	15,981.820	1065.348
25–29	16.7	14,051.044	2347.227	8.6	14,051.044	1214.853
30–34	19.1	12,102.265	2307.297	10.6	12,102.265	1285.987
35–39	21.4	9982.646	2138.782	12.6	9982.646	1258.412
40–44	23.8	7767.685	1847.544	14.6	7767.685	1132.995
45–49	26.1	6008.701	1570.975	16.6	6008.701	995.401
50–54	28.5	4993.836	1423.493	18.5	4993.836	926.157
55–59	30.9	4146.148	1279.709	20.5	4146.148	851.038
60–64	33.2	3325.733	1104.975	22.5	3325.733	748.489
65–69	35.6	2554.200	908.912	24.5	2554.200	625.421
70–74	37.9	1821.521	691.176	26.5	1821.521	482.084
75–79	40.3	1077.611	434.331	28.4	1077.611	306.537
80+	44.1	721.755	318.157	31.6	721.755	228.176
All	20.3	103,138.833	20,894.843	11.6	103,138.833	11,992.676

Note: Estimates based on epidemiologic model.

## Discussion

With about 21 million overweight and 12 million obese persons in Nigeria in 2020, Nigeria possibly represents the most affected country in Africa. Low levels of physical activity, urban drifts, unhealthy diets, socio-economic changes, and psychosocial factors are largely responsible for this high burden [55]. Many have reported that the introduction of processed foods, growth in the economy and relatively improved standards of living have resulted in fast rising rates of obesity across many Africa countries [56]. As noted, the health consequences are also fast accumulating, with increase in the prevalence of several chronic diseases, further stretching already weakened health systems in these settings. Our findings thus illuminate this burden on a national scale, hopefully prompting renewed interest and response from policy makers and stakeholders.

The age-adjusted prevalence rates of overweight and obesity in Nigeria were 20.3% and 11.3%, respectively. Our estimates are relatively similar to some previous studies, suggesting that the prevalence of obesity in Nigeria may have not changed significantly over the years. For example, in 2005, Abubakari and Bhopal [57] estimated a pooled prevalence of obesity in Nigeria at 8.8%, with this increasing to 10% in 2008 [58]. From a 2008 demographic and health survey in Nigeria, Kandala et al. [59] estimated a combined prevalence of obesity and overweight at 20.9%. In 2013, Chukwuonye et al. [60] reported that that the prevalence of overweight across Nigeria ranged from 20 to 35%, and obesity from 8 to 22%. However, Commodore-Mensah et al. [61] noted a wide range of combined prevalence of overweight and obesity in Nigeria at 4–49%, which perhaps reflects the varying demographics, geographical settings, socio-economic status and wealth index of the populations from which the data were pooled [59]. When compared to other African countries, Neupane et al. [62] reported that prevalence of overweight ranged from 6% in Madagascar to 28% Swaziland, and obesity from 1 to 23% also in the two countries. The WHO also reported that the prevalence of obesity in Sub-Saharan Africa ranges between 3.3% and 18.0% [63], which are relatively within our reported estimates for Nigeria.

The higher estimates of overweight and obesity among women compared to men that we reported are well supported by many studies. According to the 2010 WHO survey data on Nigeria, the prevalence of overweight in the country was 37% and 26%, while obesity was lower at 8% and 3%, among women and men, respectively [63]. Abubakari et al. [57] specifically noted that women were more likely to be obese than men with odds consistently between 3.2 and 4.8 across various settings in Nigeria. Some authors [13,62,64] further linked this to socio-economic status, noting that women in urban residence with higher education and wealth index had higher likelihood of being overweight or obese. Generally, across Africa, obesity appears to be a major issue among urban women aged 15–49 years, as demonstrated from the results of demographic and health survey from 24 African countries [62], with consequences being more serious as this is the average reproductive age of most women [55]. Asides the known cardio-metabolic risks, maternal obesity has resulted in higher rates of miscarriages, still births and congenital disorders [65,66].

The prevalence of overweight and obesity across the geographical regions was marked by wide uncertainties especially in the northern parts of the country, with our estimates subject to further validation. From the 2008 Nigerian demographic and health survey, Kandala et al. [59] reported striking variations in the prevalence of overweight and obesity across Nigeria ranging from 10.5% in Yobe (North-east Nigeria) to 50.2% in Lagos (South-west Nigeria). However, the South-east had highest pooled prevalence of overweight persons (33%) and one of the leading prevalence of obesity (14%) in this study. Ubesie et al. [67] reported that child obesity is major public health issue in Enugu, South-east, Nigeria, with this possibly reflecting in adolescence, young adults and the overall population over time. The authors did note that this is even more common among children of the higher socio-economic class residing in core urban settings in the city. As estimated in this study, urbanization is widely associated with increased risk of overweight and obesity. Addressing lifestyles and diet of urban dwellers is in fact a major step in the response to reducing overweight and obesity in Nigeria. Sedentary lifestyles and consumption of processed foods are on the increase in several urban settings in the country [64]. Despite seemingly high prices of processed foods, many have continued to associate with this as a way to display affluence among peers, as against the relatively cheap fruits, vegetables and whole grains [13]. In fact, higher rates of obesity appear to be correlated with national wealth status, as the epidemic of overweight and obesity is fast rising in African countries with relatively higher domestic product per capita, of which Nigeria is one [64]. As observed from number of persons affected (21 million) and the relatively high mean population BMI and waist circumference in Nigeria at 26 kg/m^2^ and 87 cm, respectively, there is need for urgent and comprehensive nationwide awareness and effective population strategies to address this growing epidemic.

Our study has some important limitations. First, heterogeneity across studies was high, which is a reflection of widespread variations in study designs, data collation, and population covered. We also observed some degree of publication bias, suggesting mainly large studies reporting high prevalence rates of obesity and overweight were getting published. Second, although our approach to quality assessment has been consistently employed in previous studies, we recognize that there are other important quality measures that could have been assessed, including missing data and response rate. Of note, we did not assess studies for standard survey guidelines (e.g. the WHO STEPwise approach to Surveillance (STEPS) of non-communicable diseases); rather, we explored case definitions as an alternative, given that prevalence rates of obesity and/or overweight were not primary focus of many studies, hence they do not necessarily employ the WHO STEPs or related protocols. Third, while the meta-regression (random-effects) accounted for sample population across the individual data-points, it could not explain (and represent) the variations across the six geopolitical zones, the 36 States and the Federal Capital Territory (FCT). Indeed, limited data point across the States, particularly in the Northern regions, meant we could only present the pooled crude estimates for these settings. For example, of the 35 studies retained, only six (17%) were from the northern parts of the country, with wide uncertainties in the pooled estimates. Lastly, although we attempted to contact authors for missing data, we only found information on study characteristics most useful in many cases, as we could not correlate several missing figures provided with published data. This also reflects in our inability to provide comprehensive estimates of BMI and waist circumference by age, sex and geographical regions. However, with 35 studies covering a population of 52,816, we believe our estimates fairly represent the epidemiology of overweight and obesity in Nigeria, and provides a robust data pool on which future studies can be based.

## Conclusions

Our findings suggest a high prevalence of overweight and obesity in Nigeria. This is marked in urban Nigeria and among women, which may in part be due to widespread sedentary lifestyles and proliferation of processed food outlets. Besides, the social status associated with these lifestyles appear to be a major factor in urban Nigeria. We call on government, policy makers, health professionals and all stakeholders to jointly work towards addressing this public health issue. There is need for population-wide awareness, health education and promotion activities relevant for home and work places, increased taxes on processed foods, and creating a conducive and safe environment for physical activity.

## Supplementary Material

Supplemental Material

## Data Availability

All underlying data in this study are included in the supplementary material. Further enquiries can be directed at the corresponding author.
