# Supplementary material for: Estimating the prevalence of overweight and obesity in Nigeria in 2020: a systematic review and meta-analysis
Source: Ann Med. 2021 Mar 30;53(1):495–507. doi: 10.1080/07853890.2021.1897665 (PMC8018557; doi:10.1080/07853890.2021.1897665)
Supplement: Supplemental Material [file IANN_A_1897665_SM9373.docx]

**SUPPLEMENTAL FILE**

**Estimating the prevalence of overweight and obesity in Nigeria in 2020: a systematic review and meta-analysis**

**Davies Adeloye**^1*^**, Janet O Ige-Elegbede^2^, Martinsixtus Ezejimofor**^3^**, Eyitayo O Owolabi^4^, Nnenna Ezeigwe^5^, Chiamaka Omoyele^5^, Rex G Mpazanje^6^, Mary T Dewan^6^, Emmanuel Agogo**^7^**, Muktar A Gadanya^8^, Wondimagegnehu Alemu^9^, Michael O Harhay**^10^**, Asa Auta**^11^**, Akindele O Adebiyi^12^**

1. Centre for Global Health, Usher Institute, University of Edinburgh, UK.
2. Faculty of Health and Applied Sciences, University of the West of England, Bristol, UK
3. Division of Health Sciences, University of Warwick, Coventry, UK
4. Centre for Global Surgery, Department of Global Health, Stellenbosch University, Stellenbosch, South Africa
5. Federal Ministry of Health, Abuja, Nigeria
6. World Health Organization, Nigeria Country Office, Abuja, Nigeria
7. Resolve to Save Lives, Abuja, Nigeria
8. Department of Community Medicine, Aminu Kano Teaching Hospital, Bayero University, Kano, Nigeria.
9. International health Consultancy, Atlanta, GA, USA
10. Department of Biostatistics, Epidemiology and Informatics, University of Pennsylvania Perelman School of Medicine, Philadelphia, PA, USA
11. School of Pharmacy and Biomedical Sciences, University of Central Lancashire, Fylde Road, Preston, UK
12. College of Medicine, University of Ibadan, Ibadan, Nigeria.

**^*^Correspondence: Dr. Davies Adeloye,** Centre for Global Health, Usher Institute, University of Edinburgh, UK. Email: davies.adeloye@ed.ac.uk

**Contents**

[Figure S1. Crude prevalence of overweight in Nigeria, men. 3](#_Toc64525691)

[Figure S2. Crude prevalence of overweight in Nigeria, women. 4](#_Toc64525692)

[Figure S3. Crude prevalence of obesity in Nigeria, men. 5](#_Toc64525693)

[Figure S4. Crude prevalence of obesity in Nigeria, women. 6](#_Toc64525694)

[Figure S5. Funnel plot 7](#_Toc64525695)

[Figure S6. Egger’s graph, showing mall study effects 8](#_Toc64525696)

[Table S1. Quality grading 9](#_Toc64525697)

[Table S2. All extracted data employed in analysis 10](#_Toc64525698)

[Table S3. Metaregression 16](#_Toc64525699)

[Overweight 16](#_Toc64525700)

[Obesity 16](#_Toc64525701)

[Retained studies 17](#_Toc64525702)

# Figure S1. Crude prevalence of overweight in Nigeria, men.

# Figure S2. Crude prevalence of overweight in Nigeria, women.

# Figure S3. Crude prevalence of obesity in Nigeria, men.

# Figure S4. Crude prevalence of obesity in Nigeria, women.

# Figure S5. Funnel plot

# Figure S6. Egger’s graph, showing mall study effects

# Table S1. Quality grading

| **Author** | **Sampling** | **Analysis** | **Ascertainment** | **Score** | **Quality** |
| --- | --- | --- | --- | --- | --- |
| 1. Abegunde & Owoaje ^1^ | 1 | 1 | 2 | 4 | High |
| 1. Agaba et al ^2^ | 1 | 1 | 2 | 4 | High |
| 1. Akinbodewa et al ^3^ | 1 | 1 | 2 | 4 | High |
| 1. Emerole et al ^4^ | 1 | 1 | 1 | 3 | Moderate |
| 1. Ibekwe ^5^ | 1 | 1 | 1 | 3 | Moderate |
| 1. Odey et al ^6^ | 1 | 1 | 1 | 3 | Moderate |
| 1. Odugbemi et al ^7^ | 1 | 1 | 2 | 4 | High |
| 1. Lawoyin et al ^8^ | 1 | 1 | 1 | 3 | Moderate |
| 1. Ugwuja et al ^9^ | 1 | 1 | 2 | 4 | High |
| 1. Oladapo et al ^10^ | 1 | 1 | 2 | 4 | High |
| 1. Okaka & Eiya ^11^ | 1 | 1 | 2 | 4 | High |
| 1. Odenigbo et al^12^ | 1 | 1 | 2 | 4 | High |
| 1. Okagua et al ^13^ | 1 | 1 | 1 | 3 | Moderate |
| 1. Adesina et al ^14^ | 1 | 1 | 1 | 3 | Moderate |
| 1. Oyeyemi & Adeyemi ^15^ | 1 | 1 | 2 | 4 | High |
| 1. Iwuala et al ^16^ | 1 | 1 | 2 | 4 | High |
| 1. Musa et al ^17^ | 1 | 1 | 1 | 3 | Moderate |
| 1. Yusuf et al ^18^ | 1 | 1 | 2 | 4 | High |
| 1. Odunaiya et al ^19^ | 1 | 1 | 2 | 4 | High |
| 1. Ezejimofor et al ^20^ | 1 | 1 | 2 | 4 | High |
| 1. Ojji et al ^21^ | 1 | 1 | 2 | 4 | High |
| 1. Akintunde et al ^22^ | 1 | 1 | 2 | 4 | High |
| 1. Akpan et al ^23^ | 1 | 1 | 2 | 4 | High |
| 1. Chukwuonye et al ^24^ | 1 | 1 | 2 | 4 | High |
| 1. Ezekwesili et al ^25^ | 1 | 1 | 2 | 4 | High |
| 1. Iloh et al ^26^ | 1 | 1 | 2 | 4 | High |
| 1. Iloh et al ^27^ | 1 | 1 | 1 | 3 | Moderate |
| 1. Iloh et al ^28^ | 1 | 1 | 1 | 3 | Moderate |
| 1. Murthy et al ^29^ | 1 | 1 | 2 | 4 | High |
| 1. Okafor et al ^30^ | 1 | 1 | 1 | 3 | Moderate |
| 1. Ogah et al ^31^ | 1 | 1 | 2 | 4 | High |
| 1. Olamoyegun et al ^32^ | 1 | 1 | 1 | 3 | Moderate |
| 1. Shittu et al ^33^ | 1 | 1 | 1 | 3 | Moderate |
| 1. Suleiman et al ^34^ | 1 | 1 | 1 | 3 | Moderate |
| 1. Wahab et al ^35^ | 1 | 1 | 2 | 4 | High |

# Table S2. All extracted data employed in analysis

| Author | Study Period | Location | Geopolitical zone | Study design | Study Setting | Population xtics | Obesity type | Mean BMI | Waist circ (cm) | Mean age | Cases (all) | Sample (all) | Prev % (all) | Age (male) | Cases (male) | Sample (male) | Prev % (male) | Age (female) | Cases (female) | Sample (female) | Prev % (female) |
| --- | --- | --- | --- | --- | --- | --- | --- | --- | --- | --- | --- | --- | --- | --- | --- | --- | --- | --- | --- | --- | --- |
| Agaba et al | 2014 | Jos, Plateau State | North-central | Descriptive cross-sectional study | Urban | Higher education institution | Obesity | 27.2 |  | 44 | 236 | 883 | 26.70 | 43 | 56 | 529 | 10.60 | 45 | 177 | 354 | 50.00 |
| Akinbodewa et al | 2014 | Akure & Ondo, Ondo State | South-west | Descriptive cross-sectional study | Mixed | General population | Overweight | 26.2 | 85.9 | 44.7 | 330 | 1183 | 27.90 |  |  |  |  |  |  |  |  |
| Akinbodewa et al | 2014 | Akure & Ondo, Ondo State | South-west | Descriptive cross-sectional study | Mixed | General population | Obesity |  |  | 44.7 | 274 | 1183 | 23.20 |  |  |  |  |  |  |  |  |
| Emerole et al | 2007 | Owerri, Imo State | South-east | Descriptive cross-sectional study | Urban | Higher education institution | Overweight |  |  | 53 | 84 | 241 | 34.85 |  |  |  |  |  |  |  |  |
| Ibekwe | 2012 | Oghara, Delta State | South-south | Descriptive cross-sectional study | Rural | General population | Obesity |  |  | 36.7 | 51 | 272 | 18.80 |  |  |  |  |  |  |  |  |
| Odey et al | 2011 | Calabar, Cross River State | South-south | Descriptive cross-sectional study | Urban | Secondary school | Obesity |  |  | 14.67 | 7 | 375 | 1.90 |  | 2 | 146 | 1.40 |  | 5 | 229 | 2.20 |
| Odugbemi et al | 2010 | Tejuosho, Lagos | South-west | Descriptive cross-sectional study | Urban | Traders | Overweight |  |  | 43.3 | 152 | 400 | 38.00 | 45.5 | 43 | 103 | 41.70 | 42.3 | 109 | 297 | 36.70 |
| Odugbemi et al | 2010 | Tejuosho, Lagos | South-west | Descriptive cross-sectional study | Urban | Traders | Obesity |  |  | 43.3 | 47 | 400 | 11.80 | 45.5 | 15 | 103 | 14.60 | 42.3 | 32 | 297 | 10.80 |
| Lawoyin et al | 1998 | Idikan Ibadan, Oyo State | South-west | Population-based cross-sectional study | Rural | General population | Obesity |  |  | 55 | 549 | 2144 | 25.60 | 55 | 236 | 892 | 26.50 | 55 | 318 | 1252 | 25.40 |
| Ugwuja et al | 2008 | Abakaliki, Ebonyi State | South-east | Descriptive cross-sectional study | Urban | Civil servants | Overweight | 24.5 |  | 40.9 | 70 | 205 | 34.20 | 40.9 | 44 | 106 | 41.50 | 40.9 | 26 | 99 | 26.30 |
| Ugwuja et al | 2008 | Abakaliki, Ebonyi State | South-east | Descriptive cross-sectional study | Urban | Civil servants | Obesity |  |  | 40.9 | 14 | 205 | 6.80 | 40.9 | 8 | 106 | 7.80 | 40.9 | 6 | 99 | 6.10 |
| Oladapo et al | 2005 | Egbeda, Oyo State | South-west | Descriptive cross-sectional study | Rural | General population | Overweight | 24.2 | 88.3 | 42.1 | 38 | 2000 | 1.90 | 42.1 | 7 | 873 | 1.90 | 42.1 | 21 | 1127 | 1.80 |
| Oladapo et al | 2005 | Egbeda, Oyo State | South-west | Descriptive cross-sectional study | Rural | General population | Obesity |  |  | 42.1 | 40 | 2000 | 2.00 | 42.1 | 13 | 873 | 1.50 | 42.1 | 27 | 1127 | 2.40 |
| Okaka & Eiya | 2013 | Ovia, Edo state | South-south | Population-based cross-sectional study | Rural | General population | Obesity |  |  | 48 | 30 | 161 | 18.60 |  |  |  |  |  |  |  |  |
| Odenigbo et al | 2008 | Asaba, Delta State | South-south | Population-based cross-sectional study | Semi-urban | General population | Obesity |  |  | 41.59 | 33 | 100 | 33.00 | 41.59 | 11 | 49 | 22.45 | 41.59 | 22 | 51 | 43.14 |
| Okagua et al | 2016 | Port-Harcourt, Rivers State | South-south | Population-based cross-sectional study | Urban | Secondary school | Overweight |  |  | 16 | 301 | 2282 | 13.20 | 16 |  |  | 11.40 | 16 |  |  | 14.60 |
| Okagua et al | 2016 | Port-Harcourt, Rivers State | South-south | Population-based cross-sectional study | Urban | Secondary school | Obesity |  |  | 16 | 105 | 2282 | 4.60 | 16 |  |  | 5.20 | 16 |  |  | 5.20 |
| Adesina et al | 2010 | Port-Harcourt, Rivers State | South-south | Population-based cross-sectional study | Urban | Secondary school | Overweight |  |  | 15 | 63 | 960 | 6.30 | 15 | 18 | 481 | 3.70 | 15 | 45 | 479 | 9.40 |
| Adesina et al | 2010 | Port-Harcourt, Rivers State | South-south | Population-based cross-sectional study | Urban | Secondary school | Obesity |  |  | 15 | 17 | 960 | 1.80 | 15 | 3 | 481 | 0.60 | 15 | 14 | 479 | 2.90 |
| Oyeyemi & Adeyemi | 2013 | Maiduguri, Yobe State | North-east | Population-based cross-sectional study | Semi-urban | General population | Overweight | 25 |  | 44.9 | 88 | 292 | 30.10 | 44.9 | 51 | 190 | 36.30 | 44.9 | 37 | 102 | 26.80 |
| Oyeyemi & Adeyemi | 2013 | Maiduguri, Yobe State | North-east | Population-based cross-sectional study | Semi-urban | General population | Obesity |  |  | 44.9 | 70 | 292 | 24.00 | 44.9 | 27 | 190 | 42.10 | 44.9 | 43 | 102 | 14.30 |
| Iwuala et al | 2014 | Lagos State | South-west | Descriptive cross-sectional study | Urban | Health workers | Overweight | 27.7 |  | 39.3 | 134 | 300 | 44.67 |  |  |  |  |  |  |  |  |
| Iwuala et al | 2014 | Lagos State | South-west | Descriptive cross-sectional study | Urban | Health workers | Obesity |  |  | 39.3 | 82 | 300 | 27.33 |  |  |  |  |  |  |  |  |
| Musa et al | 2012 | Benue State | North-central | Descriptive cross-sectional study | Mixed | Secondary school | Overweight |  |  | 16 | 216 | 2226 | 9.70 |  |  |  |  |  |  |  |  |
| Musa et al | 2012 | Benue State | North-central | Descriptive cross-sectional study | Mixed | Secondary school | Obesity |  |  | 16 | 40 | 2226 | 1.80 |  |  |  |  |  |  |  |  |
| Yusuf et al | 2013 | Kano State | North-west | Descriptive cross-sectional study | Urban | Secondary school | Overweight |  |  | 16 | 14 | 718 | 1.98 | 16 | 4 | 309 | 1.29 |  | 10 | 409 | 2.44 |
| Yusuf et al | 2013 | Kano State | North-west | Descriptive cross-sectional study | Urban | Secondary school | Obesity |  |  | 16 | 6 | 718 | 0.84 | 16 | 2 | 309 | 0.65 |  | 4 | 409 | 0.98 |
| Odunaiya et al | 2010 | Ibadan, Oyo State | South-west | Population-based cross-sectional study | Urban | Secondary school | Overweight |  |  | 16.17 | 88 | 1000 | 8.80 |  |  |  |  |  |  |  |  |
| Odunaiya et al | 2010 | Ibadan, Oyo State | South-west | Population-based cross-sectional study | Urban | Secondary school | Obesity |  |  | 16.17 | 12 | 1000 | 1.20 |  |  |  |  |  |  |  |  |
| Ezejimofor et al | 2014 | Niger Delta, Delta State | South-south | Community-based cross-sectional study | Rural | General population | Overweight |  |  | 44.32 | 786 | 2028 | 38.76 |  |  |  |  |  |  |  |  |
| Ezejimofor et al | 2014 | Niger Delta, Delta State | South-south | Community-based cross-sectional study | Rural | General population | Obesity |  |  | 44.32 | 331 | 2028 | 16.32 |  |  |  |  |  |  |  |  |
| Ojji et al | 2010 | Abuja, FCT | North-central | Prospective cohort study | Urban | General population | Obesity | 27.5 |  | 49.1 | 409 | 1515 | 27.00 | 49.9 | 136 | 747 | 18.20 | 48.1 | 263 | 768 | 34.20 |
| Akintunde et al | 2010 | Osogbo, Osun State | South-west | Population-based cross-sectional study | Mixed | General population | Overweight | 26.5 |  | 54.97 | 286 | 816 | 35.05 | 54.97 | 160 | 420 | 38.10 | 54.97 | 126 | 396 | 31.82 |
| Akintunde et al | 2010 | Osogbo, Osun State | South-west | Population-based cross-sectional study | Mixed | General population | Obesity |  |  | 54.97 | 208 | 816 | 25.49 | 54.97 | 71 | 420 | 16.90 | 54.97 | 137 | 396 | 34.60 |
| Akpan et al | 2015 | Akwa Ibom States | South-south | Population-based cross-sectional study | Urban | General population | Obesity |  |  | 39.9 |  | 590 | 27.80 | 39.9 |  | 155 |  | 39.9 |  | 435 |  |
| Akpan et al | 2015 | Akwa Ibom States | South-south | Population-based cross-sectional study | Rural | General population | Obesity |  |  | 43.9 |  | 978 | 7.20 | 43.9 |  | 292 |  | 43.9 |  | 686 |  |
| Chukwuonye et al | 2013 | Abia State | South-east | Population-based house-to-house survey | Mixed | General population | Overweight | 25.2 | 85.7 | 41.95 | 684 | 2807 | 24.37 | 41.95 | 335 | 1377 | 24.33 | 41.95 | 349 | 1429 | 24.42 |
| Chukwuonye et al | 2013 | Abia State | South-east | Population-based house-to-house survey | Mixed | General population | Obesity |  |  | 41.95 | 312 | 2807 | 11.12 | 41.95 | 91 | 1377 | 7.73 | 41.95 | 221 | 1429 | 14.37 |
| Ezekwesili et al | 2016 | Anambra State | South-east | Population-based cross-sectional study | Mixed | General population | Overweight |  |  | 38 | 311 | 912 | 34.10 |  |  |  |  |  |  |  |  |
| Ezekwesili et al | 2016 | Anambra State | South-east | Population-based cross-sectional study | Mixed | General population | Obesity |  |  | 38 | 127 | 912 | 13.93 |  |  |  |  |  |  |  |  |
| Iloh et al | 2009 | Imo State | South-east | Descriptive cross-sectional study | Rural | General population | Obesity |  |  | 40 | 129 | 2156 | 6.00 |  |  |  |  |  |  |  |  |
| Iloh et al | 2008 | Imo State | South-east | Descriptive cross-sectional study | Rural | General population | Obesity |  |  | 37 | 684 | 9296 | 7.40 |  |  |  |  |  |  |  |  |
| Iloh et al | 2010 | Owerri, Imo State | South-east | Descriptive cross-sectional study | Urban | General population | Obesity |  |  | 42 | 206 | 2391 | 8.60 |  |  |  |  |  |  |  |  |
| Murthy et al | 2013 | National | National | Population-based cross-sectional study | Mixed | General population | Overweight |  |  | 55.9 | 2588 | 13504 | 19.16 | 56.8 |  | 6203 |  | 55.2 |  | 7301 |  |
| Murthy et al | 2013 | National | National | Population-based cross-sectional study | Mixed | General population | Obesity |  |  | 55.9 | 1113 | 13504 | 8.24 | 56.8 |  | 6203 |  | 55.2 |  | 7301 |  |
| Okafor et al | 2014 | Enugu, Enugu State | South-east | Population-based cross-sectional study | Urban | General population | Overweight |  |  | 49.1 | 295 | 775 | 38.10 | 50.8 | 96 | 279 | 34.40 | 48.2 | 199 | 496 | 40.10 |
| Okafor et al | 2014 | Enugu, Enugu State | South-east | Population-based cross-sectional study | Urban | General population | Obesity |  |  | 49.1 | 164 | 775 | 21.20 | 50.8 | 44 | 279 | 15.80 | 48.2 | 120 | 496 | 24.20 |
| Ogah et al. 2013 | 2012 | Umuahia, Abia State | South-east | Population-based cross-sectional study | Mixed | General population | Obesity |  |  | 41.7 | 1006 | 2983 | 33.70 | 41.5 | 431 | 1430 | 30.10 | 41.8 | 575 | 1553 | 37.00 |
| Olamoyegun et al | 2016 | Ekiti State | South-west | Population-based cross-sectional study | Semi-urban | General population | Overweight | 23.4 | 85.7 | 61.7 | 161 | 750 | 21.50 | 60 | 44 | 218 | 19.90 | 62.4 | 117 | 542 | 22.10 |
| Olamoyegun et al | 2016 | Ekiti State | South-west | Population-based cross-sectional study | Semi-urban | General population | Obesity |  |  | 61.7 | 64 | 750 | 8.50 | 60 | 5 | 218 | 2.30 | 62.4 | 59 | 542 | 11.20 |
| Shittu et al | 2017 | Oke Ogun, Oyo State | South-west | Population-based cross-sectional study | | General population | Overweight |  |  | 55.2 | 600 | 6915 | 8.68 |  |  |  |  |  |  |  |  |
| Shittu et al | 2017 | Oke Ogun, Oyo State | South-west | Population-based cross-sectional study | | General population | Obesity |  |  | 55.2 | 705 | 6915 | 10.20 |  |  |  |  |  |  |  |  |
| Suleiman et al. 2013 | 2011 | Amassoma, Bayelsa State | South-south | Descriptive cross-sectional study | Semi-urban | General population | Overweight |  |  | 50.5 | 126 | 400 | 31.50 |  |  |  |  |  |  |  |  |
| Suleiman et al. 2013 | 2011 | Amassoma, Bayelsa State | South-south | Descriptive cross-sectional study | Semi-urban | General population | Obesity |  |  | 50.5 | 61 | 400 | 15.25 |  |  |  |  |  |  |  |  |
| Wahab et al | 2006 | Katsina, Katsina State | North-west | Population-based cross-sectional study | Urban | General population | Overweight |  |  | 37.6 | 160 | 300 | 53.30 | 38 | 54 | 129 | 41.90 | 37.2 | 106 | 171 | 62.00 |
| Wahab et al | 2006 | Katsina, Katsina State | North-west | Population-based cross-sectional study | Urban | General population | Obesity |  |  | 37.6 | 63 | 300 | 21.00 | 38 | 12 | 129 | 9.30 | 37.2 | 51 | 171 | 29.80 |

# Table S3. Metaregression

## Overweight

## Obesity

# Retained studies

1. Abegunde KA, Owoaje ET. Health problems and associated risk factors in selected urban and rural elderly population groups of South-West Nigeria. *Annals of African Medicine* 2013; **12**: 90-7.

2. Agaba EI, Akanbi MO, Agaba PA, et al. A survey of non-communicable diseases and their risk factors among university employees: a single institutional study. *Cardiovascular journal of Africa* 2017; **28**: 377-84.

3. Akinbodewa A, Adejumo A, Koledoye O, et al. Community screening for pre-hypertension, traditional risk factors and markers of chronic kidney disease in Ondo State, South-Western Nigeria. *Nigerian Postgraduate Medical Journal* 2017; **24**: 25-30.

4. Emerole CO, Aguwa EN, Onwasigwe CN, Nwakoby BA. Cardiac risk indices of staff of Federal University Of Technology Owerri, Imo State, Nigeria. *Tanzania health research bulletin* 2007; **9**: 132-5.

5. Ibekwe R. Modifiable Risk factors of Hypertension and Socio-demographic Profile in Oghara, Delta State; Prevalence and Correlates. *Annals of medical and health sciences research* 2015; **5**: 71-7.

6. Odey FA, Okokon IB, Ogbeche JO, Jombo G, Ekanem E. Prevalence of cigarette smoking among adolescents in Calabar city, south-eastern Nigeria. *Journal of Medicine and Medical Sciences* 2012; **3**: 237-42.

7. Odugbemi TO, Onajole AT, Osibogun AO. Prevalence of cardiovascular risk factors amongst traders in an urban market in Lagos, Nigeria. *Nigerian Postgraduate Medical Journal* 2012; **19**: 1-6.

8. Lawoyin TO, Asuzu MC, Kaufman J, et al. Prevalence of cardiovascular risk factors in an African, urban inner city community. *West African journal of medicine* 2002; **21**: 208-11.

9. Ugwuja E, Ezenkwa U, Nwibo A, Ogbanshi M, Idoko O, Nnabu R. Prevalence and determinants of hypertension in an agrarian rural community in southeast Nigeria. *Annals of medical and health sciences research* 2015; **5**: 45-9.

10. Oladapo OO, Salako L, Sodiq O, Shoyinka K, Adedapo K, Falase AO. A prevalence of cardiometabolic risk factors among a rural Yoruba south-western Nigerian population: a population-based survey. *Cardiovascular Journal of Africa* 2010; **21**: 26-31.

11. Okaka EI, Okwuonu CG. Blood pressure variation and its correlates among patients undergoing hemodialysis for renal failure in Benin City, Nigeria. *Annals of African medicine* 2017; **16**: 65-9.

12. Odenigbo CU, Oguejiofor OC. Pattern of medical admissions at the Federal Medical Centre, Asaba-a two year review. *Nigerian journal of clinical practice* 2009; **12**: 395-7.

13. Okagua J, Alex-Hart BA, Jaja TP. Overweight and obesity status of school adolescents in Portharcourt, southern Nigeria. *Nigerian journal of medicine : journal of the National Association of Resident Doctors of Nigeria* 2016; **25**: 53-9.

14. Adesina AF, Peterside O, Anochie I, Akani NA. Weight status of adolescents in secondary schools in port Harcourt using Body Mass Index (BMI). *Italian journal of pediatrics* 2012; **38**: 31.

15. Oyeyemi AL, Adeyemi O. Relationship of physical activity to cardiovascular risk factors in an urban population of Nigerian adults. *Archives of public health = Archives belges de sante publique* 2013; **71**: 6.

16. Iwuala SO, Ayankogbe OO, Olatona FA, et al. Obesity among health service providers in Nigeria: danger to long term health worker retention? *The Pan African medical journal* 2015; **22**: 1.

17. Musa DI, Toriola AL, Monyeki MA, Lawal B. Prevalence of childhood and adolescent overweight and obesity in Benue State, Nigeria. *Tropical medicine & international health : TM & IH* 2012; **17**: 1369-75.

18. Yusuf S, Mijinyawa M, Musa B, Gezawa I, Uloko A. Overweight and obesity among adolescents in Kano, Nigeria. *J Metab Syndr* 2013; **2**: 126.

19. Odunaiya NA, Grimmer K, Louw QA. High prevalence and clustering of modifiable CVD risk factors among rural adolescents in southwest Nigeria: implication for grass root prevention. *BMC public health* 2015; **15**: 661.

20. Ezejimofor MC, Uthman OA, Maduka O, et al. The Burden of Hypertension in an Oil- and Gas-Polluted Environment: A Comparative Cross-Sectional Study. *American journal of hypertension* 2016; **29**: 925-33.

21. Ojji DB, Ajayi SO, Mamven MH, Alabi P. Prevalence of metabolic syndrome among hypertensive patients in Abuja, Nigeria. *Ethnicity & disease* 2012; **22**: 1-4.

22. Akintunde AA, Akinwusi PO, Adebayo RA, Ogunyemi S, Opadijo OG. Burden of obesity in essential hypertension: pattern and prevalence. *Nigerian journal of clinical practice* 2010; **13**: 399-402.

23. Akpan EE, Ekrikpo UE, Udo AI, Bassey BE. Prevalence of Hypertension in Akwa Ibom State, South-South Nigeria: Rural versus Urban Communities Study. *International journal of hypertension* 2015; **2015**: 975819.

24. Chukwuonye, II, Chuku A, Onyeonoro UU, et al. Prevalence of abdominal obesity in Abia State, Nigeria: results of a population-based house-to-house survey. *Diabetes, metabolic syndrome and obesity : targets and therapy* 2013; **6**: 285-91.

25. Ezekwesili CN, Ononamadu CJ, Onyeukwu OF, Mefoh NC. Epidemiological survey of hypertension in Anambra state, Nigeria. *Nigerian journal of clinical practice* 2016; **19**: 659-67.

26. Iloh GU, Ikwudinma AO, Obiegbu NP. Obesity and Its Cardio-metabolic Co-morbidities Among Adult Nigerians in a Primary Care Clinic of a Tertiary Hospital in South-Eastern, Nigeria. *Journal of family medicine and primary care* 2013; **2**: 20-6.

27. Iloh G, Amadi AN, Nwankwo BO, Ugwu VC. Obesity in adult Nigerians: a study of its pattern and common primary co-morbidities in a rural Mission General Hospital in Imo state,South-Eastern Nigeria. *Nigerian journal of clinical practice* 2011; **14**: 212-8.

28. Iloh GU, Amadi AN, Nwankwo BO. Obesity in adult Nigerians: a study of its prevalence and common primary co-morbidities in a semi-urban Mission General Hospital in South-Eastern Nigeria. *Nigerian journal of medicine : journal of the National Association of Resident Doctors of Nigeria* 2010; **19**: 459-66.

29. Murthy GV, Fox S, Sivasubramaniam S, et al. Prevalence and risk factors for hypertension and association with ethnicity in Nigeria: results from a national survey. *Cardiovascular journal of Africa* 2013; **24**: 344-50.

30. Okafor CI, Gezawa ID, Sabir AA, Raimi TH, Enang O. Obesity, overweight, and underweight among urban Nigerians. *Nigerian journal of clinical practice* 2014; **17**: 743-9.

31. Ogah OS, Madukwe OO, Onyeonoro UU, et al. Cardiovascular risk factors and non-communicable diseases in Abia state, Nigeria: report of a community-based survey. *Int J Med Biomed Res* 2013; **2**: 57-68.

32. Olamoyegun MA, Oluyombo R, Iwuala SO, Asaolu SO. Epidemiology and patterns of hypertension in semi-urban communities, south-western Nigeria. *Cardiovascular journal of Africa* 2016; **27**: 356-60.

33. Shittu RO, Odeigah LO, Fakorede KO, et al. Prevalence and correlates of hypertension-outcome of a free medical screening in Oke-Ogun area of Oyo state, Nigeria, West Africa. *Journal of the American Society of Hypertension : JASH* 2018; **12**: 268-74.

34. Suleiman IA, Amogu EO. Prevalence of hypertension in Amassoma, Southern Ijaw, Bayelsa state, Nigeria. *Value in Health* 2012; **Conference: 17th Annual International Meeting of the International Society for Pharmacoeconomics and Outcomes Research, ISPOR 2012 Washington, DC United States. Conference Start: 20120602 Conference End: 20120606. Conference Publication: (var.pagings). 15 (4)**: A116.

35. Wahab KW, Sani MU, Yusuf BO, Gbadamosi M, Gbadamosi A, Yandutse MI. Prevalence and determinants of obesity - a cross-sectional study of an adult Northern Nigerian population. *International archives of medicine* 2011; **4**: 10.
